# Induction chemotherapy with nedaplatin, docetaxel and 5-fluorouracil followed by concurrent nedaplatin and radiotherapy in locoregionally advanced nasopharyngeal carcinoma: A single arm, open label, phase II clinical trial

**DOI:** 10.1016/j.tranon.2025.102634

**Published:** 2026-01-06

**Authors:** Fang-Zheng Chen, Ying Deng, Wen-Jing Yin, Meng-Yao Wang, Fang Yang, Zhi-Huan Yang, Li-Ping Zhou, Si-Da Chen, Jie-Ling Chen, Xi-Zhen Jiang, Ao-Xiong Zhou, Yu-Meng Ou, Jin-Quan Liu, Dong-Ping Chen, Bin Qi

**Affiliations:** a Guangzhou Institute of Cancer Research, the Affiliated Cancer Hospital, Guangzhou Medical University, Guangzhou 510095, China; b State Key Laboratory of Oncology in South China, Guangdong Key Laboratory of Nasopharyngeal Carcinoma Diagnosis and Therapy, Guangdong Provincial Clinical Research Center for Cancer, Sun Yat-Sen University Cancer Center, Guangzhou 510060, China; c Hospital of Chengdu Office of People’s Government of Tibetan Autonomous Region (Hospital. C.T.), No. 20, Xi Mian Qiao Heng Jie, Wuhou District, Chengdu 610041, Sichuan, China

**Keywords:** Nasopharyngeal carcinoma, Nedaplatin, Induction chemotherapy, Concurrent chemotherapy, Radiotherapy, Chemotherapy, Chemoradiotherapy

## Abstract

**Purpose:**

To evaluate the efficacy of nedaplatin in induction chemotherapy and concurrent chemoradiotherapy for locoregionally advanced nasopharyngeal carcinoma.

**Methods:**

In this prospective, single-arm, open-label phase II trial, patients with newly diagnosed stage III-IVa (except T3–4N0) nasopharyngeal carcinoma were enrolled. Participants received three cycles of induction chemotherapy with docetaxel (60 mg/m² IV on days 1, 22, and 43), nedaplatin (60 mg/m² IV on days 1, 22, and 43), and fluorouracil (600 mg/m²/day as a continuous 120 h infusion on days 1–5, 22–26, and 43–47). This was followed by intensity-modulated radiotherapy with concurrent nedaplatin (100 mg/m² IV on days 1, 22, and/or 43) for two or three cycles. The primary endpoint was the objective response rate (ORR). Secondary endpoints included overall survival (OS), progression-free survival (PFS), and toxicity profile.

**Results:**

From March 2020 to November 2021, 32 patients were enrolled. With a median follow-up of 42.4 months (IQR, 35.4–45.2), 32 patients (100 %) achieved ORR at 12 weeks post-treatment. The 36-month PFS was 87.5 % (95 % CI, 76.1 %-98.8 %), and the 36-month OS was 100 %. The most common grade 3 or 4 adverse events during induction chemotherapy were neutropenia (9.4 %), diarrhea (9.4 %), leukopenia (6.2 %), fatigue (3.1 %) and hepatotoxicity (3.1 %). Mucositis (9.4 %) was the most common adverse events during concurrent chemoradiotherapy, followed by leukopenia (3.1 %), neutropenia (3.1 %), and thrombocytopenia (3.1 %). All adverse events were manageable.

**Conclusion:**

Induction chemotherapy with nedaplatin, docetaxel, and 5-fluorouracil, followed by concurrent nedaplatin with intensity-modulated radiotherapy, demonstrated promising antitumor activity and manageable toxicities in locoregionally advanced nasopharyngeal carcinoma patients.

**Trial registration:**

This trial was registered with ClinicalTrials.gov identifier: NCT04834206.

## Highlights

• Nedaplatin works well for both induction and concurrent chemotherapy treatments.
• Nedaplatin, docetaxel, and 5-FU form an effective induction chemotherapy regimen.
• Nedaplatin treatment offers reliable objective response rates and survival outcomes.
• Nedaplatin is well-tolerated with predictable, manageable side effects.
• Nedaplatin is a viable alternative to cisplatin in treating nasopharyngeal carcinoma.

## Introduction

Nasopharyngeal carcinoma (NPC), arising from epithelial cells of the nasopharynx, exhibits a distinctive geographical prevalence, particularly in southern China and Southeast Asia [1]. Induction chemotherapy (IC) followed by concurrent chemoradiotherapy (CCRT) has been validated as an effective treatment strategy in clinical trials [[2], [3], [4]] and is recommended by clinical guidelines [5,6] for locoregionally advanced NPC (LA-NPC), which is present in about 70 % of newly diagnosed patients.

Cisplatin based regimen is commonly the first choice for IC or CCRT. Sun et al. reported docetaxel-cisplatin-5-fluorouracil (TPF) as an efficacy and safe IC regimen [2,3]. Despite the absence of head-to-head trials comparing various IC regimens, TPF has demonstrated commendable long-term survival outcomes statistically [3]. Nevertheless, the clinical utility of cisplatin is constrained by its toxicities, particularly gastrointestinal, nephrotoxic, and neurotoxic effects. Complex hydration and antiemetic requirements further extended hospital stays, increased costs, and challenges in adherence to cisplatin treatment [7,8]. It is of paramount importance to have an effective, less toxic, well-tolerated and convenient alternative to cisplatin.

Nedaplatin, a second-generation platinum analog, disrupts DNA duplication, offering a potentially alternative to cisplatin with less toxic and potential synergistic in combination with radiation [9]. Its mild adverse reactions and convenient delivery enhance treatment compliance and quality of life compared to cisplatin [10]. Several clinical trials have validated nedaplatin as a viable substitute for cisplatin in the treatment for LA-NPC [[11], [12], [13], [14], [15]].

Encouraged by the promising antitumor effects and favorable safety profile of nedaplatin, we embarked on this phase II trial, aiming to explore its substitution for cisplatin in both TPF induction chemotherapy and concurrent chemotherapy regimens for LA-NPC.

## Materials and methods

### Study design and participants

This prospective single-arm phase II trial was conducted at Guangzhou Institute of Cancer Research, the Affiliated Cancer Hospital, Guangzhou Medical University, China, and was registered with ClinicalTrials.gov (NCT04834206). The trial adhered to the principles outlined in the Declaration of Helsinki and the International Conference on Harmonization of Good Clinical Practice. The trial protocol received approval from the ethics review board of Guangzhou Institute of Cancer Research, the Affiliated Cancer Hospital, Guangzhou Medical University. Prior to enrollment, written informed consents were obtained from all participating patients.

Eligibility criteria included the following: age between 18 and 65 years; histologically or cytologically confirmed as nonkeratinizing nasopharyngeal carcinoma (including differentiated and undifferentiated subtypes, WHO II or III); newly diagnosed stage III to IVa disease (except T3–4N0) according to the American Joint Committee on Cancer/Union for International Cancer Control (AJCC/UICC) 8th edition stage-classification system; no evidence of distant metastasis; Eastern Cooperative Oncology Group performance score of 0–1; adequate hematologic function (white blood cell count ≥ 4 × 10^9^/L, platelet count ≥ 100 × 10^9^/L, and hemoglobin ≥ 90 g/L); adequate renal function (creatinine clearance ≥ 60 mL/min); and adequate hepatic function (serum bilirubin, alanine aminotransferase, and aspartate aminotransferase ≤ 2.0 times the upper limit of normal). Exclusion criteria were the following: receipt of treatment with palliative intent; invasive malignancies except adequately treated basal cell or squamous cell skin cancer, or *in situ* cervical cancer; receipt of previous surgery (except diagnostic) or chemotherapy for primary tumors or nodes; lactation or pregnancy; or severe intercurrent disease (*i.e.*, unstable cardiac disease requiring treatment, acute exacerbation of chronic obstructive pulmonary disease or other respiratory illness requiring admission to hospital, active hepatitis, and mental disturbance).

### Pretreatment evaluation

Essential pretreatment evaluations included the following: complete patient history; physical examination; hematology and biochemistry profiles; plasma Epstein-Barr virus (EBV) DNA load within two weeks before enrollment; electrocardiography; fiber optic nasopharyngoscopy; histopathological diagnosis; enhanced magnetic resonance imaging (MRI) or enhanced computed tomography (CT) of the nasopharynx and neck (CT was indicated only in patients with contraindication to MRI). Whole-body 18F-fluorodeoxyglucose positron emission tomography-CT (^18^F-FDG PET-CT) was recommended for the detection of distance metastasis. Enhanced CT of the chest and abdomen and whole-body skeletal scintigraphy with technetium-99 m methylene diphosphonate (Tc-MDP) were optional substitution to PET-CT. All patients were referred for dental examination before radiotherapy.

### Procedures

Eligible patients underwent three cycles of docetaxel-nedaplatin-5-fluorouracil (TNF) IC followed by CCRT. TNF IC regimen comprised docetaxel 60 mg/m² intravenously every 3 weeks on days (D) 1, 22 and 43, nedaplatin 60 mg/m² intravenously every 3 weeks on D 1, 22 and 43, and fluorouracil 600 mg/m² per day as a continuous 120 h infusion every 3 weeks on D 1–5, 22–26 and 43–47. Concurrent nedaplatin was administered 3 weeks after the start of the last cycle of IC in intervals. Nedaplatin was intravenously administered more than 1 hour at a dose of 100 mg/m^2^ on D 1, 21 and /or 43, coincided with IMRT for two or three cycles. The determination of administering two or three cycles of concurrent chemotherapy (CCT) was based on a collective decision by the investigators, considering treatment benefits and patient tolerability. Nedaplatin is of moderate emetic risk, prophylactically antiemetics were administered according to the guidelines [16]. In case of grade 3 or higher adverse reactions, chemotherapy was postponed until recovery. Subsequently, the dose of the next chemotherapy cycle was reduced by 20 %. If grade 3 or higher adverse effects persisted after two reductions, chemotherapy was discontinued. If unpredicted situations such as nedaplatin-related severe hypersensitivity reactions arise during the whole treatment, cisplatin will be given in the following treatment. Granulocyte colony-stimulating factor (G-CSF), pegylated recombinant human granulocyte-colony stimulating factor (PEG-rhG-CSF), or recombinant human interleukin-11 (rhIL-11) were employed for myelosuppression. Platelet transfusions could be used to treat severe thrombocytopenia or spontaneous bleeding caused by thrombocytopenia.

Intensity-modulated radiotherapy (IMRT) was mandatory in all patients. The target volumes were contoured according to the International Commission on Radiation Units and Measurements (ICRU) Reports 50 and 62. The prescribed radiation doses for planning target volume (PTV)nx, PTVnd, PTV1 and PTV2 were 66–70 Gy, 64–70 Gy, 60–62 Gy and 54–56 Gy, respectively, in 30–33 fractions, delivered daily at five fractions per week over 6–7 weeks. Dose constraints to the critical structures were within the tolerance according to the Radiation Therapy Oncology Group (RTOG) 0225 protocol and our former study [11,17]. Patients were removed from the study if they had disease progression or severe comorbidities during treatment or withdrew consent at any time during the study.

Patients underwent follow-up every 3 months in the initial 3 years post-treatment and then at 6-month intervals until death. Follow-up assessments comprised a physical examination, enhanced MR or CT of the head and neck, fiber-optic nasopharyngoscopy, chest radiograph or CT scan, ultrasonography or CT scan of the entire abdomen, and quantitative determination of plasma EBV DNA. Whole-body skeletal scintigraphy or PET-CT was conducted as necessary, at the discretion of the investigators. Acute and late toxicities associated with chemoradiotherapy were assessed according to the established National Cancer Institute Common Terminology Criteria for Adverse Events (NCI-CTCAE) version 5.0, RTOG and European Organization for Research and Treatment of Cancer (EORTC) late radiation morbidity scoring scheme [18].

Target lesions underwent evaluation based on the Response Evaluation Criteria in Solid Tumors Version 1.1 (RECIST V1.1) criteria, recording instances of complete response (CR), partial responses (PR), stable disease (SD), and progressive disease (PD), along with noting the specific time points at which the status changes occurred.

Biopsy to the suspicious lesions was recommended to confirm the disease progress. If histologic evidence were not easily accessible, a clinical diagnosis would be given if presenting with persistent indicative imaging features and symptoms. Otherwise, subsequent follow-up would be performed closely to ascertain the status of the equivocal imaging findings. Salvage treatments including re-irradiation, chemotherapy, or surgery were provided in cases of documented relapse or residual disease if possible.

For patients lost to follow-up or alive without distant metastasis or locoregional recurrence at the trial's conclusion, their data were censored at the last follow-up date.

### Study endpoints

The primary endpoint was objective response rate (ORR), defined as the sum of CR and PR based on Response Evaluation Criteria in Solid Tumors Version 1.1 (RECIST V1.1) criteria at twelve weeks post-radiotherapy. Secondary endpoints included overall survival (OS), defined as the duration from the initiation of treatment to death from any cause; progression-free survival (PFS), defined as the time from treatment onset to locoregional failure, distant failure, or death from any cause, whichever occurred first; distant metastasis-free survival (DMFS), defined as the duration from treatment commencement to distant metastasis or death from any cause; local recurrence-free survival (LRFS), defined as the time from treatment initiation to local or regional relapse or death from any cause; and safety.

### Sample size determination and statistical analysis

The sample size determination followed Simon’s two-stage design, aiming for a study power of 0.9 and a two-sided significance level of 0.05 to detect treatment failure. Assuming an ORR of 90 %, with less than 70 % deemed unacceptable, accounting for a 10 % dropout rate, a total of 32 patients were planned for enrollment. The PowerAndSampleSize tool (https://powerandsamplesize.com/Calculators/) guided this estimation. In stage one, among 18 evaluable patients, if the number of responders was less than 13, the study would be terminated. Conversely, the study enters stage two, an additional 14 patients would be enrolled.

Efficacy analyses were conducted in the intention-to-treat population, while the safety analysis included all patients who received at least one cycle of protocol treatment, regardless of their eligibility status. Patient characteristics, safety data, and anti-tumor activity were descriptively summarized. The median follow-up time was calculated using the reverse Kaplan-Meier method. The 95 % CIs of the ORR was determined by the Clopper and Pearson method. OS, PFS, LRFS and DMFS were plotted using the Kaplan-Meier method. The relative dose intensities of drugs represent the ratio of the doses administered to the doses specified in the protocol. Statistical analyses were performed using R software (version 4.3.2) and SPSS (version 25.0).

## Results

### Patients characteristics

The baseline demographics and disease characteristics are listed in Table 1. All patients were histologically confirmed as WHO type III NPC. Twenty-four patients (75.0%) were diagnosed with stage III, and 8 (25.0%) were diagnosed with stage IVa.

**Table 1:** Baseline patient demographics and clinical characteristics.

Characteristic	Patients (N = 32)
Age, years	
Median (Range)	44 (21, 57)
Sex, No. ( %)	
Female	6 (18.8)
Male	26 (81.2)
Histology, No. ( %)	
WHO III	32 (100.0)
Karnofsky performance status score, No. ( %)	
90	32 (100.0)
T category, No. ( %)	
T1	1 (3.1)
T2	6 (18.8)
T3	21 (65.2)
T4	4 (12.5)
N category, No. ( %)	
N0	0 (0)
N1	11 (34.3)
N2	16 (50.0)
N3	5 (15.6)
TNM Stage, No. ( %)	
III	24 (75.0)
IVa	8 (25.0)
Pre-treatment EBV DNA, copies/mL	
Median (Range)	1946 (0, 697,800)
<4000 copies/ml, No. ( %)	20 (62.5)
≥4000 copies/ml, No. ( %)	12 (37.5)

NOTE. T category and N category refer to the T stage and N stage, respectively, according to the TNM staging system (American Joint Committee on Cancer/Union for International Cancer Control, 8th edition).

Abbreviation: EBV, Epstein-Barr virus.

**Fig. 1:**
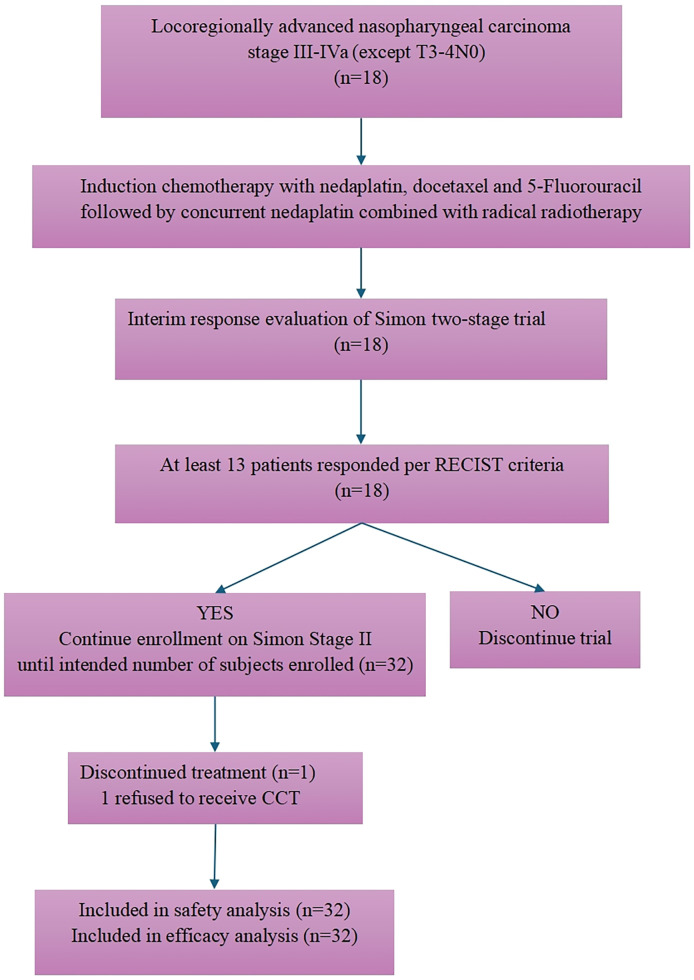
Flowchart of the trial.

Abbreviation: RECIST, Response Evaluation Criteria in Solid Tumors; CCT, concurrent chemotherapy.

The initial 13 patients enrolled achieved CR or PR, which satisfied 13 out of the 18 criteria, leading to the expansion of the trial into its second stage. Totally, 32 patients entered the study between March 2020 and November 2021. All patients were included in the efficacy and safety analysis (Fig. 1).

### Compliance and Chemoradiotherapy delivery

All 32 (100 %) patients started TNF IC. During the IC course, 1 (3.1 %) patient got grade 3 hepatotoxicity after the first cycle and discontinued the following ICs. Other 31 (96.9 %) patients completed protocol-defined three cycles of IC. Dose reductions by one level were reported in 5 (16.1 %) patients, mainly due to grade 3–4 hematological toxic effects. No two levels or more dose reduction happened. Treatment delay was observed in 1 (3.1 %) patient during IC, due to grade 1 hepatotoxicity and postponed for 4 days. The mean relative dose intensities were 97.4 % for docetaxel, 97.4 % for nedaplatin, and 96.4 % for fluorouracil. The median duration of ICs (from the first day of cycle 1 to the last day of cycle 3) was 47.0 days (Range, 5–52). The median duration from the beginning of IC to the beginning of RT was 64.0 days (Range, 26–74) (Appendix Table S1 and Fig S1, online only).

Thirty-two (100.0 %) patients started the protocol defined CCRT and completed the scheduled total radiation dose. The median duration of radiotherapy was 47.0 days (Range, 45–69). The median dose per fraction was 2.12 Gy (Range, 2.12–2.26), and the median RT dose was 70 Gy (Range, 70–76). Nine (28.1 %) of 32 patients received 3 cycles of CCT. Thirty-one (96.9 %) patients completed two cycles of CCT, whereas the remaining one patient did not receive CCT due to personal refusal. As a result, 5 (15.6 %) patients received 300 mg/m^2^ concurrent nedaplatin, 26 (81.3 %) patients received at least 200 mg/m² concurrent nedaplatin, and 5 (15.6 %) patients received between 100 mg/m² and 200 mg/m² concurrent nedaplatin. The mean relative dose intensity for concurrent nedaplatin was 71.5 % (Appendix Table S2 and Fig S1, online only).

### Survival outcomes

Thirty-two (100 %) patients achieved objective responses (CR or PR) after ICs before the start of CCRT. Of these responders, 16 (50.0 %) had CR, 16 (50.0 %) had PR. No one got SD or PD.

After the completion of CCRT, all 32 patients (100 %) experienced ORR, included 26 (81.3 %) CRs and 6 (18.6 %) PRs. Of these 6 partial responders, all had residual disease in lymph nodes.

**Fig. 2:**
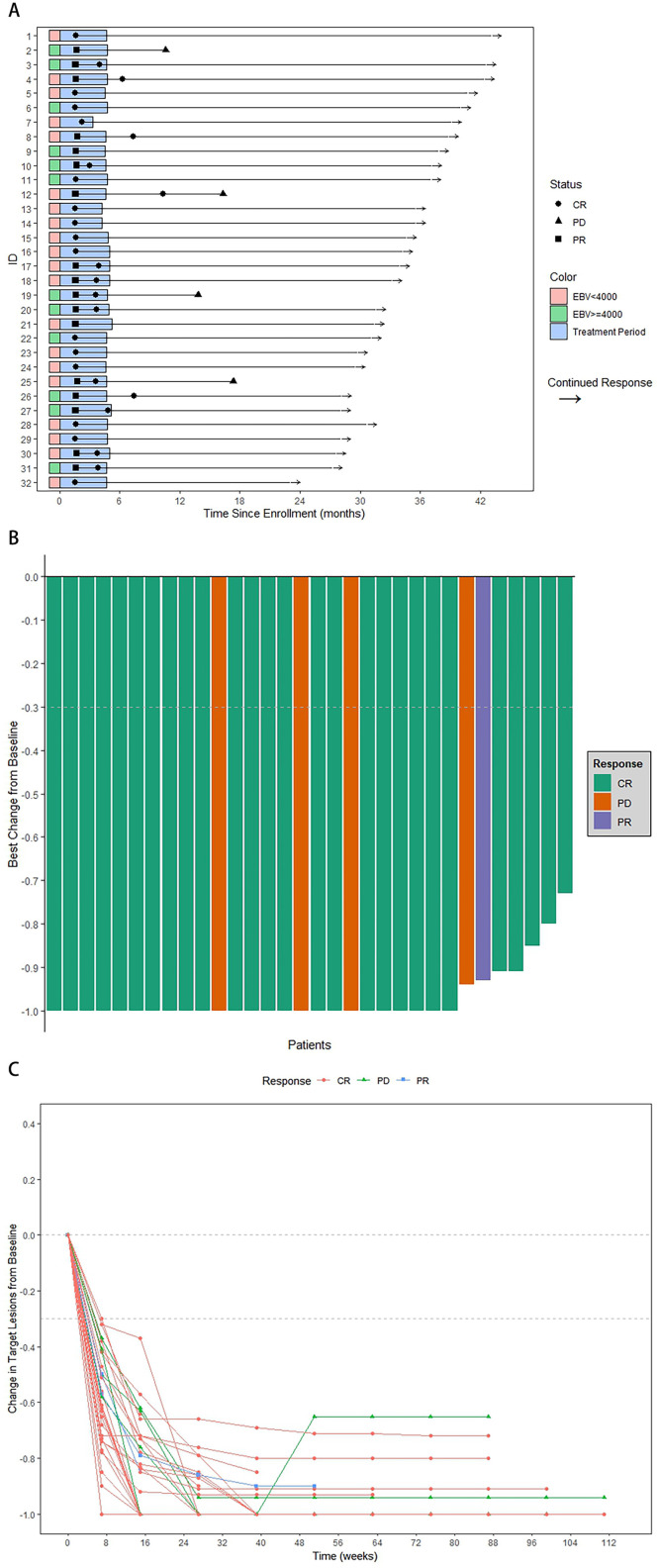
The Waterfall plot, Spider plot and Swimmer plot of patients (N = 32) with locally advanced nasopharyngeal carcinoma in the study. (A) The length of each bar represents the duration of treatment for each patient. The length of the straight line represents the length of the follow-up period. (B) Change from baseline in total size of tumor target lesion for each patient according to Response Evaluation Criteria in Solid Tumors Version 1.1 (RECIST V1.1) criteria. (C) Maximum change from baseline in total size of tumor target lesion according to RECIST V1.1 criteria.

Abbreviations: CR, complete response; PR, partial response; SD, stable disease; PD, progressive disease; EBV, Epstein-Barr virus.

All 32 patients (100.0 %) had post-treatment assessment. All target lesions decreased in the sizes. At 12 weeks after treatment, 31 (96.9 %) patients achieved CRs, one (3.1 %) patient remained PR, and no patients were evaluated as SD nor PD. (Table 2 and Fig. 2).

**Table 2:** Treatment responses in 32 patients.

Treatment response	After IC No. ( %)	After CCRT No. ( %)	Twelve weeks after CCRT No. ( %)	Last follow-up No. ( %)
CR	16/32 (50.0)	26/32 (81.3)	31/32 (96.9)	25/32(78.1)
PR	16/32 (50.0)	6/32 (18.8)	1/32 (3.1)	1/32(3.1)
SD	0/32 (0)	0/32 (0)	0/32 (0)	0/32 (0)
PD	0/32 (0)	0/32 (0)	0/32 (0)	4/32 (12.5)
ORR (CR+PR)	32/32 (100)	32/32 (100)	32/32 (100)	28/32(87.5)
95 % CI of ORR	(1.000, 1.000)	(1.000, 1.000)	(1.000, 1.000)	(0.761,0.988)

Abbreviations: IC, induction chemotherapy; CCRT, concurrent chemotherapy; CR, complete response; PR, partial response; SD, stable disease; PD, progressive disease; ORR, objective response rate; CI, confidence interval.

**Fig. 3:**
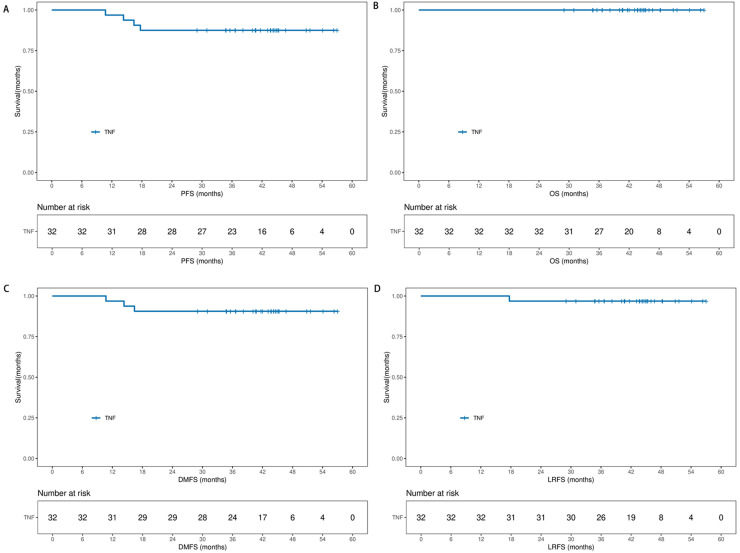
Kaplan-Meier curves of the progression-free survival (PFS), overall survival (OS), distant metastasis-free survival (DMFS) and local recurrence-free survival (LRFS). (A) The PFS, (B) OS, (C) DMFS and (D) LRFS were assessed in the intent to treat population (n = 32).

The data cutoff date for safety and efficacy analysis was January 1, 2025, with a median follow-up of 42.4 months (IQR, 35.4–45.2). None lost follow-up, 4 (12.5 %) got progression event, and 28 (87.5 %) were disease free alive. Two patients (p01002&p01026) got oligometastase in liver at 7.4 and 10.6 months after treatment, one patient (p01017) got oligometastase in third thoracic vertebra at 13.1 months after treatment and one patient (p01036) got nasopharyngeal recurrence at 14.1 months after treatment. The 36-month PFS was 87.5 % (95 % CI, 76.1 % to 98.8 %) and the 36-month OS was 100 %. The median OS and PFS did not reach (Fig. 3).

To investigate the association between baseline EBV-DNA and efficacy, patients were categorized into low (<4000 copies/mL, *n* = 20) and high (≥4000 copies/mL, *n* = 12) EBV-DNA groups. The ORR was 100 % in both groups. However, the 3-year PFS was 83.3 % (95 % CI, 48.2 % to 95.6 %) for the high EBV-DNA group and 90.0 % (95 % CI, 65.6 % to 97.4 %) for the low EBV-DNA group, indicating a potential association between higher baseline EBV-DNA and poorer long-term outcomes, albeit not statistically significant with the current sample size (*p* = 0.54)(Appendix Fig S2, online only).

### Toxicity

All 32 patients were included in the safety analysis. During the IC phase, the most common adverse events (AEs) were grade 1 or 2 anemia (18 [56.2 %]), leukopenia (18 [56.2 %]), neutropenia (18 [56.2 %]), nausea (16 [50 %]), hepatoxicity (15 [46.9 %]), diarrhea (11 [34.4 %]) and fatigue (11 [34.4 %]). Grade 3 or 4 neutropenia, diarrhea occurred in 3 (9.4 %) patients, followed by leukopenia (2 [6.3 %]), hepatoxicity (3.1 %) and fatigue (1 [3.1 %]) (Table 3). One patient got grade 3 hepatoxicity after the first cycle of IC. The following ICs were cancelled, and the hepatic function recovered.

**Table 3:** Acute adverse events in safety population (N = 32).

Adverse Events	No. ( %) of patients by toxicity grade during treatment				
	All grades	Grade 1–2	Grade 3	Grade 4	Grade 5
During IC					
Hematologic, No. ( %)					
Leukopenia	20 (62.5)	18 (56.2)	2 (6.3)	0 (0)	0 (0)
Neutropenia	21 (65.6)	18 (56.2)	2 (6.3)	1 (3.1)	0 (0)
Anemia	18 (56.2)	18 (56.2)	0 (0)	0 (0)	0 (0)
Thrombocytopenia	1 (3.1)	1 (3.1)	0 (0)	0 (0)	0 (0)
Nonhematologic, No. ( %)					
Nausea	16 (50.0)	16 (50.0)	0 (0)	0 (0)	0 (0)
Vomiting	1 (3.1)	1 (3.1)	0 (0)	0 (0)	0 (0)
Diarrhea	14 (43.8)	11 (34.4)	3 (9.4)	0 (0)	0 (0)
Mucositis	3 (9.4)	3 (9.4)	0 (0)	0 (0)	0 (0)
Hepatoxicity	16 (50.0)	15 (46.9)	1 (3.1)	0 (0)	0 (0)
Allergic reaction	3 (9.4)	3 (9.4)	0 (0)	0 (0)	0 (0)
Rash	6 (18.8)	6 (18.8)	0 (0)	0 (0)	0 (0)
Fatigue	12 (37.5)	11 (34.4)	1 (3.1)	0 (0)	0 (0)
During CCRT					
Hematologic, No. ( %)					
Leukopenia	20 (62.5)	19 (59.4)	1 (3.1)	0 (0)	0 (0)
Neutropenia	12 (37.5)	11 (34.4)	1 (3.1)	0 (0)	0 (0)
Anemia	24 (75.0)	24 (75.0)	0 (0)	0 (0)	0 (0)
Thrombocytopenia	6 (18.8)	5 (15.6)	1 (3.1)	0 (0)	0 (0)
Nonhematologic, No. ( %)					
Nausea	18 (56.3)	18 (56.3)	0 (0)	0 (0)	0 (0)
Vomiting	8 (25.0)	8 (25.0)	0 (0)	0 (0)	0 (0)
Anorexia	23 (71.9)	23 (71.9)	0 (0)	0 (0)	0 (0)
Constipation	5 (15.6)	5 (15.6)	0 (0)	0 (0)	0 (0)
Diarrhea	0 (0)	0 (0)	0 (0)	0 (0)	0 (0)
Hiccups	4 (12.5)	4 (12.5)	0 (0)	0 (0)	0 (0)
Weight loss	2 (6.25)	2 (6.25)	0 (0)	0 (0)	0 (0)
Mucositis	31 (96.9)	28 (87.5)	3 (9.4)	0 (0)	0 (0)
Dysphagia	1 (3.1 %)	1 (3.1 %)	0 (0)	0 (0)	0 (0)
Dermatitis	31 (96.9)	31 (96.9)	0 (0)	0 (0)	0 (0)
Allergic reaction	4 (12.5)	4 (12.5)	0 (0)	0 (0)	0 (0)
Rash	0 (0)	0 (0)	0 (0)	0 (0)	0 (0)
Pain	31 (96.9)	31 (96.9)	0 (0)	0 (0)	0 (0)
Fatigue	10 (31.3)	10 (31.3)	0 (0)	0 (0)	0 (0)
Fever	1 (3.1)	1 (3.1)	0 (0)	0 (0)	0 (0)
Hemorrhage	8 (25)	8 (25)	0 (0)	0 (0)	0 (0)
Sensory neuropathy	0 (0)	0 (0)	0 (0)	0 (0)	0 (0)
Auditory and/or hearing	3 (9.4)	3 (9.4)	0 (0)	0 (0)	0 (0)
Dry mouth	31 (96.9)	31 (96.9)	0 (0)	0 (0)	0 (0)
Hypokalemia	5 (15.6)	5 (15.6)	0 (0)	0 (0)	0 (0)
Hyponatremia	3 (9.4)	3 (9.4)	0 (0)	0 (0)	0 (0)
Hypocalcemia	4 (12.5)	4 (12.5)	0 (0)	0 (0)	0 (0)
Creatine increase	1 (3.1)	1 (3.1)	0 (0)	0 (0)	0 (0)
Hypoalbuminemia	9 (28.1)	9 (28.1)	0 (0)	0 (0)	0 (0)
Total bilirubin increase	0 (0)	0 (0)	0 (0)	0 (0)	0 (0)
ALT increase	1 (3.1)	1 (3.1)	0 (0)	0 (0)	0 (0)
AST increase	0 (0)	0 (0)	0 (0)	0 (0)	0 (0)

NOTE. Data are No. ( %). Abbreviations: IC, induction chemotherapy; CCRT, concurrent chemoradiotherapy; ALT, alanine aminotransferase; AST, aspartate aminotransferase.

During the CCRT phase, 4 (12.5 %) patients had grade 3 or 4 AEs. The most common grade 3 or 4 AEs were mucositis (3 [9.4 %]), leukopenia (1 [3.1 %]), neutropenia (1 [3.1 %]) and thrombocytopenia (1 [3.1 %]).

During the entire treatment course, one patient developed grade 4 neutropenia during IC phase. No grade 5 AE or treatment-related death events were reported in our study (Table 3). Thrombocytopenia was reported in 6 (18.8 %) patients. One (3.1 %) patient was grade 3, other 5 (15.6 %) were grade 1–2. No patient received platelet infusion (Appendix Fig S3, online only).

## Discussion

Cisplatin, a stalwart in the treatment of NPC for decades, has demonstrated its ability to significantly enhance the efficacy of radiotherapy. The cisplatin-based triplet IC, combined with cisplatin-IMRT-based CCRT, exhibited superior clinical outcomes in a multi-center clinical trial[2,3]. Consequently, it earned the status of a first-line treatment recommendation for patients with LA-NPC in the clinical guidelines[5,6]. However, the considerable toxicity and limited patient tolerance associated with cisplatin prompted further exploration of alternative options, leading to the investigation of carboplatin, lobaplatin, and nedaplatin as potential substitutes for cisplatin [11,12,19,20].

Nedaplatin, a second-generation platinum complex, stands out for its reduced gastrointestinal and renal toxicities compared to cisplatin. Additionally, it offers the advantage of not requiring high fluid-level infusions, making its delivery more convenient. In a multi-center phase 3 clinical trial, in which we participated, nedaplatin was compared to cisplatin in CCRT for LA-NPC patients. Patients treated with nedaplatin achieved a non-inferior 2-year PFS compared to cisplatin (89.9 % *vs.* 88.0 %) and experienced lower rates of vomiting, anorexia, electrolyte disturbance, hearing impairment, and renal dysfunction [11,12]. These findings suggested the viability of incorporating nedaplatin into the IC regimen. To our knowledge, the present trial is the first to evaluate the efficacy and safety of a nedaplatin-based triplet IC regimen followed by nedaplatin-IMRT-based CCRT in LA-NPC.

The present study did not include T3–4N0 subgroup, aligning with the approach of several randomized controlled trials that have assessed induction or adjuvant chemotherapy in LA-NPC. This exclusion is consistent with the understanding that these patients, with lower risk and better outcomes, do not gain additional benefits from extra chemotherapy [2,[21], [22], [23]]. The ORRs reached 93.8 % after completing IC and 100 % after the entire treatment course. These outcomes align with those observed in previous studies involving cisplatin-based IC followed by CCRT in the IMRT era [2,21,22,[24], [25], [26]].

Thirty-one patients (96.9 %) successfully completed the three prescribed courses of IC. Only one patient discontinued after the first IC cycle due to grade 3 hepatoxicity. This dropout rate is comparable to the study involving the TPF IC regimen [2]. As for concurrent chemotherapy, 22 patients (68.8 %) completed two courses, including the patient who received only one IC. Nine patients (28.1 %) successfully finished the third course of concurrent chemotherapy. Unlike the TPF study, our protocol allowed flexibility regarding the third course of chemotherapy, and the decision was made by the investigators. The decision to omit the third concurrent chemotherapy was influenced by factors such as time, chemotherapy side effects, tumor evaluations, and individual clinical experience. Nearing the end of treatment, patients often faced compromised health conditions, hypoalimentation due to oropharyngeal mucositis, and concerns about cumulative acute toxicities. Importantly, satisfactory tumor image evaluations and EBV quantities obviated the need for additional chemotherapy.

The ORRs were comparable between the two- and three-course subgroups, as the high ORRs achieved after IC were sustained. In a phase II randomized controlled trial, a two-cycle 100mg/m^2^ concurrent cisplatin regimen was found to be non-inferior to a three-cycle 100mg/m^2^ regimen in low-risk patients [27]. The dose of concurrent chemotherapy could potentially be further reduced with the incorporation of IC. An ongoing case-controlled phase 3 study has hypothesized that similar efficacy can be attained by eliminating concurrent chemotherapy when combined with IC and IMRT. Preliminary results support this research hypothesis [28].

The predominant adverse events during IC were grade 1 or 2 myelosuppression and gastrointestinal toxicities. Grade 3 or 4 neutropenia and diarrhea were observed in 3 (9.4 %) patients, and these adverse events promptly recovered with immediate intervention. No severe toxicities, other than hematological toxicities, were reported during IC. CCRT induced adverse events related to radiotherapy, such as dermatitis (31 [96.9 %]), pain (31 [96.9 %]), dry mouth (31 [96.9 %]), and mucositis (28 [87.5 %]), which were predominantly graded 1 or 2. Grade 3 or 4 hematologic toxicities during CCRT included leukopenia (1 [3.1 %]), neutropenia (1 [3.1 %]), and thrombocytopenia (1 [3.1 %]), all of which were uncomplicated and manageable.

Hematologic adverse events were special concerned by the investigators, especially thrombocytopenia. Thrombocytopenia occurred in 3.1 % during IC and 18.8 % during CCRT, slightly lower than reported in other studies [11,29]. Severe thrombocytopenia (39×10^9/L) was observed in one patient after three cycles of IC and one cycle of concurrent chemotherapy, resolving without complications.

Compared to the historical published data[2], nedaplatin-based TNF regimen demonstrated a comparable 3-year PFS (87.5 % *vs.* 80 %). However, the distinguishing feature of the TNF regimen lies in its substantially improved safety profile. The incidences of grade 3–4 neutropenia (12.5 % *vs.* 41 %) and leukopenia (9.2 % *vs.* 42 %) during IC in our study were markedly lower than those reported with TPF. Furthermore, while the TPF regimen was associated with considerable rates of grade 3–4 vomiting (22 %) and nausea (19 %) during the entire treatment course, our TNF regimen resulted in predominantly grade 1–2 gastrointestinal events, with no grade 3–4 vomiting or nausea observed during IC. This favorable toxicity profile is consistent with the known properties of nedaplatin, which offers reduced both hematological and gastrointestinal toxicities, thereby enhancing patient convenience and potentially improving treatment compliance.

Since 2019, gemcitabine plus cisplatin (GP)-based systemic treatment regimens have been increasingly reported with favorable outcomes and have now become newly recommended options in clinical guidelines [22,30,31]. In prior studies, the GP regimen demonstrated an ORR of 97.9 %, a 3-year PFS of 85.3 %, and a grade 3–4 adverse event rate of 75.7 % [22]. In trials combining GP with sintilimab (an immune checkpoint inhibitor), the ORR reached 94 %, 3-year PFS was 86 %, while the incidence of grade 3–4 adverse events remained at 74 % [31]. In the present study, TNF-based regimen achieved comparable efficacy and safety profiles, with an ORR of 100 %, 3-year PFS of 87.5 %, and a grade 3–4 adverse event rate of 40.6 %. These results indicate that the TNF regimen is comparable to both standalone GP and GP-sintilimab regimens in terms of both efficacy and adverse reaction profiles. However, these findings require further validation in larger phase III controlled trials.

This study has limitations because it was a single-arm study from single institution. A further phase III multi-center case-controlled trial (NCT04437329) is underway now, and the results are worth waiting.

## Conclusions

In conclusion, the use of nedaplatin, docetaxel, and 5-fluorouracil in IC, followed by concurrent nedaplatin alongside radical IMRT radiotherapy, demonstrated promising antitumor activity with manageable toxicities in patients with LA-NPC.

## CRediT authorship contribution statement

**Fang-Zheng Chen:** Writing – original draft. **Ying Deng:** Writing – original draft, Validation. **Wen-Jing Yin:** Writing – original draft, Investigation. **Meng-Yao Wang:** Writing – original draft, Investigation, Funding acquisition. **Fang Yang:** Writing – original draft, Investigation. **Zhi-Huan Yang:** Investigation, Data curation. **Li-Ping Zhou:** Investigation, Data curation. **Si-Da Chen:** Investigation. **Jie-Ling Chen:** Investigation. **Xi-Zhen Jiang:** Investigation. **Ao-Xiong Zhou:** Investigation. **Yu-Meng Ou:** Investigation. **Jin-Quan Liu:** Writing – review & editing, Supervision. **Dong-Ping Chen:** Writing – review & editing, Funding acquisition. **Bin Qi:** Writing – review & editing, Supervision, Funding acquisition, Conceptualization.

## Declaration of competing interest

The authors declare that they have no known competing financial interests or personal relationships that could have appeared to influence the work reported in this paper.

## Appendix Supplementary materials

Image, application 1
