# Supplementary material for: Induction chemotherapy with nedaplatin, docetaxel and 5-fluorouracil followed by concurrent nedaplatin and radiotherapy in locoregionally advanced nasopharyngeal carcinoma: A single arm, open label, phase II clinical trial
Source: Transl Oncol. 2026 Jan 6;64:102634. doi: 10.1016/j.tranon.2025.102634 (PMC12813063; doi:10.1016/j.tranon.2025.102634)
Supplement: Supplementary file 1 [file mmc1.docx]

Supplementary Appendix

Contents

Table S1 Compliance to IC 2

Table S2 Compliance to CCRT 3

Fig S1 Mean Relative Dose Intensity4

Fig S2 Kaplan-Meier curves of the progression-free survival (PFS) in EBV-DNA<4000 group and EBV-DNA≥4000 group5

Fig S3 Spider plot showed platelet level of each patient6

**Table S1:** Compliance to IC

| Variable | Patients  (N = 32) |
| --- | --- |
| Patients completing at least one cycle IC, No. (%) | 32 (100) |
| Patients completing at least two cycles IC, No. (%) | 31 (96.9) |
| Patients completing three cycles IC, No. (%) | 31 (96.9) |
| Patients completing at least two cycles IC with dose reductions, No. (%) | 5 (16.1) |
| Reason for dose modification, No. (%) |  |
| Hematologic | 2 (6.5) |
| Non-hematologic | 1 (3.2) |
| Both hematologic and non-hematologic | 2 (6.5) |
| Patients with at least one cycle delay > 3 days, No. (%) | 1 (3.1) |
| Patients with at least one cycle delay > 7 days, No. (%) | 0 (0) |
| Median (Range) duration of IC (days) | 47 (5-52) |
| Median (Range) interval between last day of IC and first day of radiotherapy (days) | 16 (7-32) |
| Median (Range) interval between first day of IC to first day of radiotherapy (days) | 64 (26-74) |

NOTE. The duration of IC was from the beginning of the first cycle of IC to the last day of the third cycle of IC.

Abbreviations: IC, induction chemotherapy.

**Table S2:** Compliance to CCRT

| Variable | Patients  (N = 32) |
| --- | --- |
| Patients receiving RT, No. (%) | 32 (100) |
| Patients completing RT, No. (%) | 32 (100) |
| Median (Range) dose of RT (Gy) | 70 (70-76) |
| Patients receiving fraction dose of 2-2.2 Gy, No. (%) | 31 (96.9) |
| Patients receiving fraction dose of >2.2 to ≤2.27 Gy, No. (%) | 1 (3.1) |
| Median (Range) dose per fraction (Gy) | 2.12 (2.12-2.26) |
| Median (Range) duration of RT (days) | 47.4 (45-49) |
| Patients starting concurrent nedaplatin, No. (%) | 31 (96.9) |
| Patients receiving at least one cycle CCT, No. (%) | 31 (96.9) |
| Patients receiving at least two cycles CCT, No. (%) | 31 (96.9) |
| Patients receiving three cycles CCT, No. (%) | 9 (28.1) |
| Patients receiving concurrent nedaplatin ≥100 mg/m^2^, <200 mg/m^2^ | 5 (15.6) |
| Patients receiving concurrent nedaplatin ≥200 mg/m^2^ | 26 (81.3) |
| Patients receiving concurrent nedaplatin 300 mg/m^2^ | 5 (15.6) |
| Median (Range) dose of CCT (mg/m^2^) | 200 (0-300) |

Abbreviations: CCRT, concurrent chemoradiotherapy; RT, radiotherapy; CCT, concurrent chemotherapy.


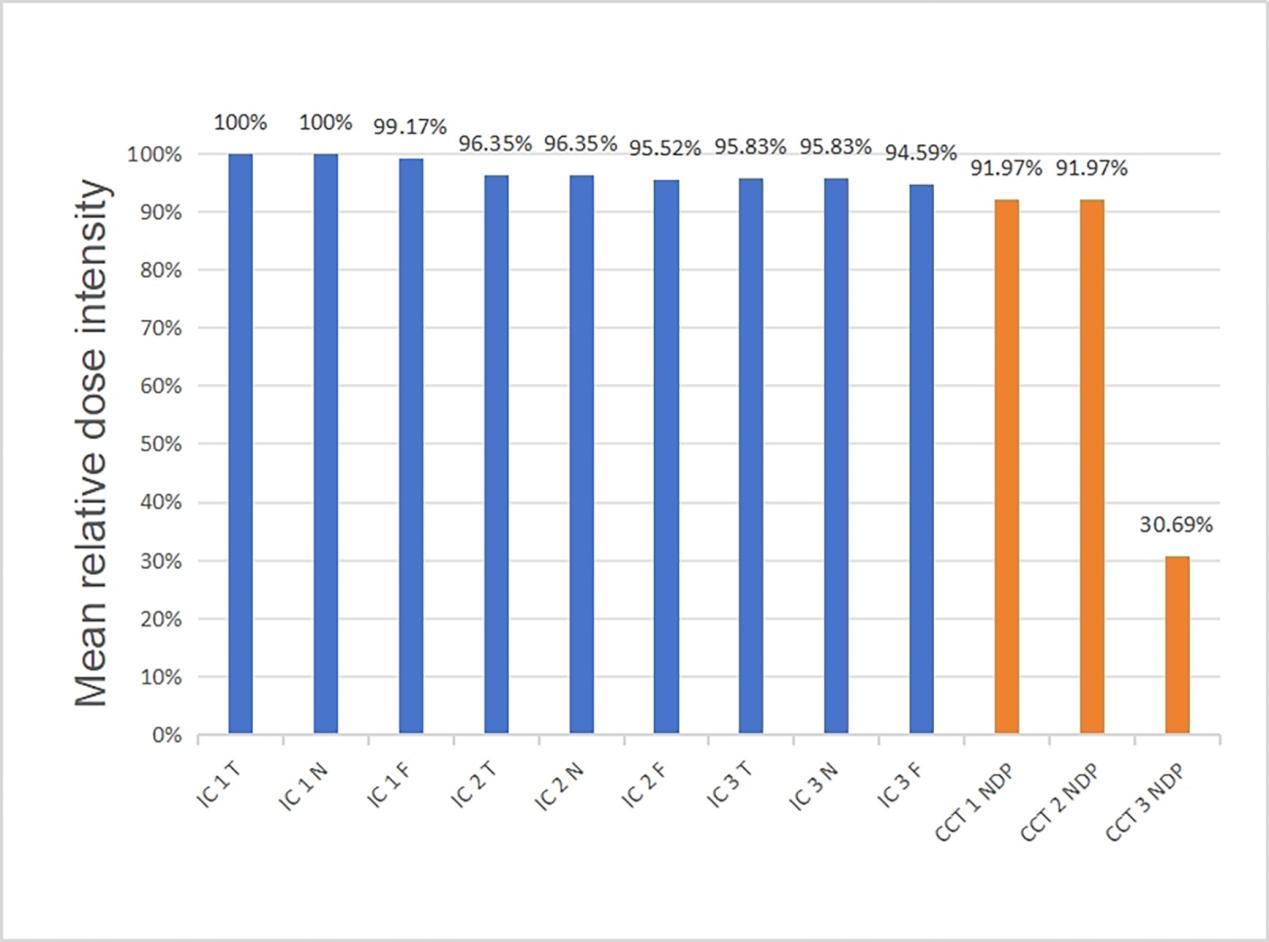


**Fig S1:** Mean Relative Dose Intensity

Abbreviations: IC, chemotherapy; CCT, concurrent chemotherapy; T = docetaxel 60 mg/m^2^; N = nedaplatin 60 mg/m^2^; F = fluorouracil 600 mg/m^2^/day d1-5; NDP = nedaplatin 100 mg/m^2^.


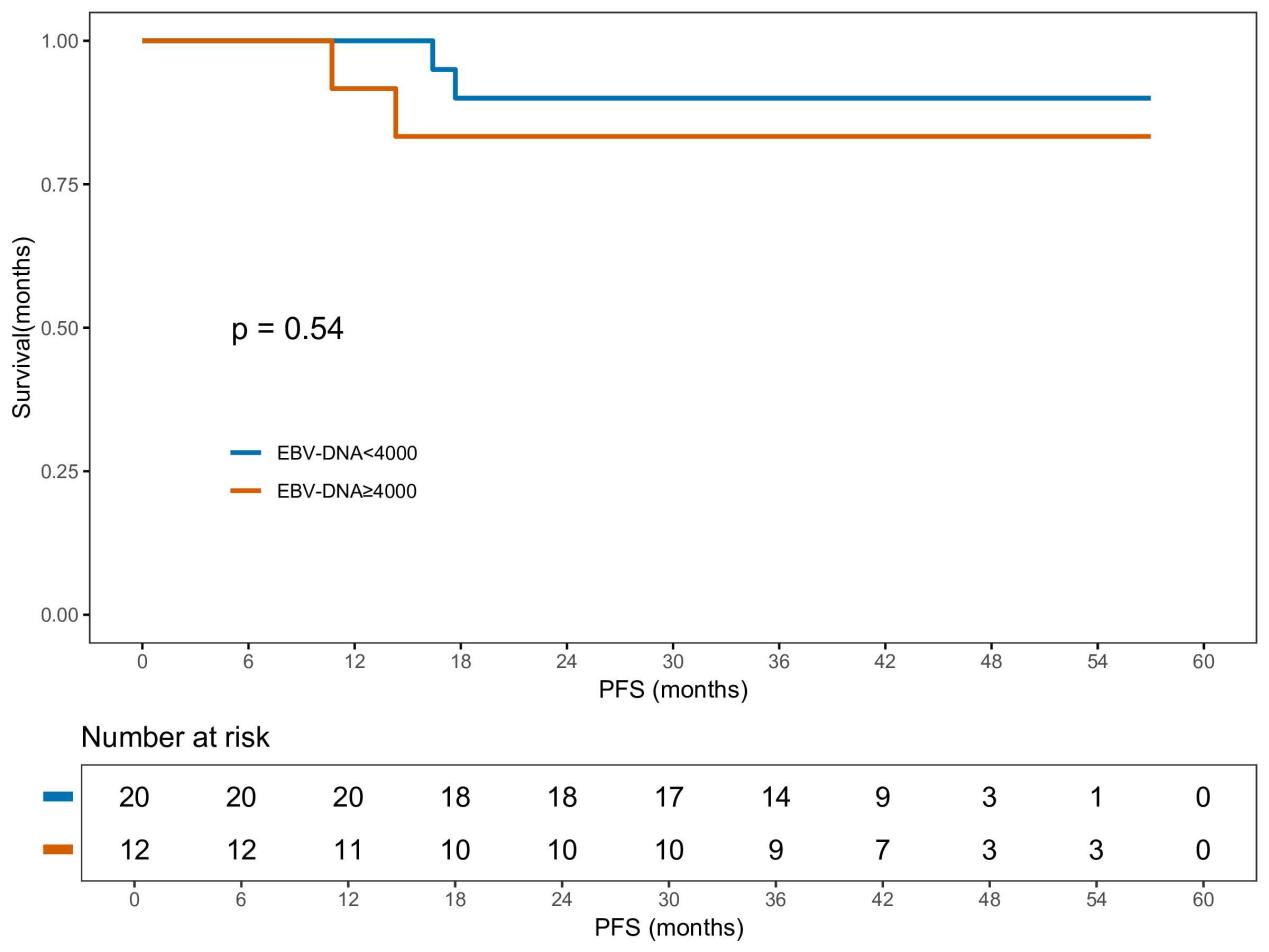


**Fig S2:** Kaplan-Meier curves of the progression-free survival (PFS) in EBV-DNA<4000 group (N=20) and EBV-DNA≥4000 group (N=12).

Abbreviations: PFS, progression-free survival; EBV, Epstein-Barr virus.


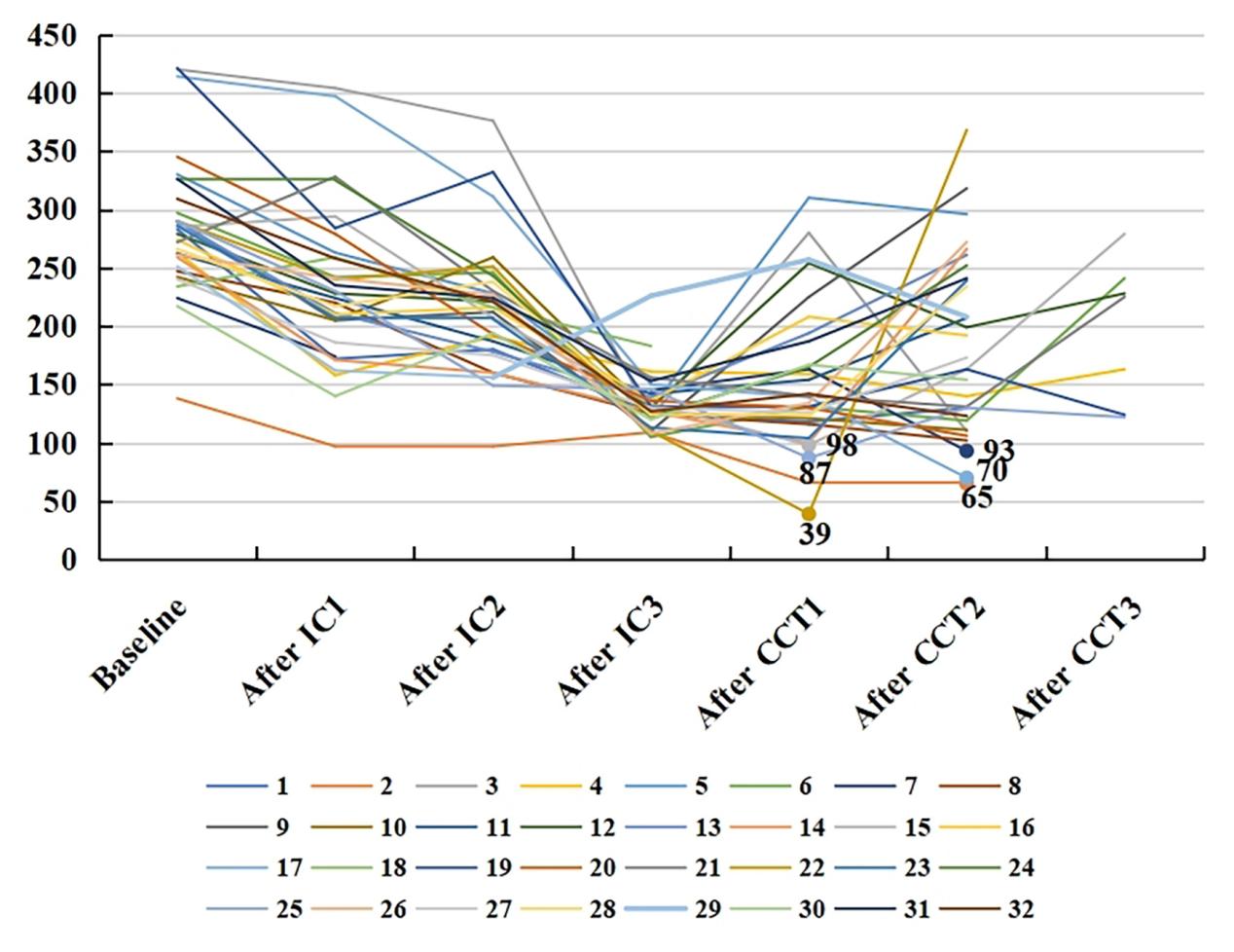


**Fig S3:** The Spider plot of patients (N=32) with locally advanced nasopharyngeal carcinoma in the study.The lines represent changes in the patient's platelet profile over the course of treatment.

Abbreviations: IC, chemotherapy; CCT, concurrent chemotherapy.
